# A Survey on End-of-Life Contemplation Among Patients on Dialysis

**DOI:** 10.1016/j.ekir.2024.07.035

**Published:** 2024-08-03

**Authors:** Martin Russwurm, Anetta Rabaev, Joachim D. Hoyer, Christian S. Haas, Christian Volberg, Philipp Russ

**Affiliations:** 1 Division of Nephrology, Centre for Internal Medicine, University Hospital Marburg, Philipps University Marburg, Marburg, Germany; 2 Institute of Pharmacology, Philipps University Marburg, Marburg, Germany; 3 Department of Anesthesiology and Intensive Care Medicine, University Hospital Marburg, Philipps University Marburg, Marburg, Germany; 4 Research Group Medical Ethics, Faculty of Medicine, Philipps University Marburg, Marburg, Germany

**Keywords:** conservative kidney care, death, end-of-life care, hemodialysis, palliative care

## Abstract

**Introduction:**

Considering that mortality among patients on dialysis is high, it would be advisable for patients, relatives, and care givers to acknowledge that after dialysis initiation for many patients, the last phase in life has begun. We sought to investigate the frequency of precautionary planning directives, contemplation about the end-of-life (EOL) and embedding of patients’ wishes in the interaction with relatives and the treating nephrologists.

**Methods:**

In a questionnaire-based interview survey, we investigated the frequency of precautionary planning, EOL wishes, and frequency of relatives’ or medical professionals’ conversations with patients about those wishes as well as possibly associated demographic, socioeconomic and medical factors. The interviews were conducted by a single investigator in 7 dialysis centers in Germany.

**Results:**

From 349 identified patients, 268 (77%) participated. The participants (36% female) had a median age of 70 (interquartile range [IQR]: 58–80) years and had spent a median of 3 (IQR: 1–7.5) years on dialysis. Overall, 46% of patients on dialysis contemplated their EOL wishes at least occasionally. Of those, 85% talked about EOL wishes with their relatives, whereas 19% discussed them with their nephrologists, yet another 28% would like to have such a discussion with their nephrologist.

**Conclusion:**

Almost half of patients on dialysis contemplate their EOL and the vast majority engage in discussions about that with their relatives. Despite patients being interested, the frequency of consultation of nephrologists on EOL care is low. This study suggests that there is a substantial but unmet need for EOL care consultation for patients on dialysis.

## Graphical abstract

**Figure:**
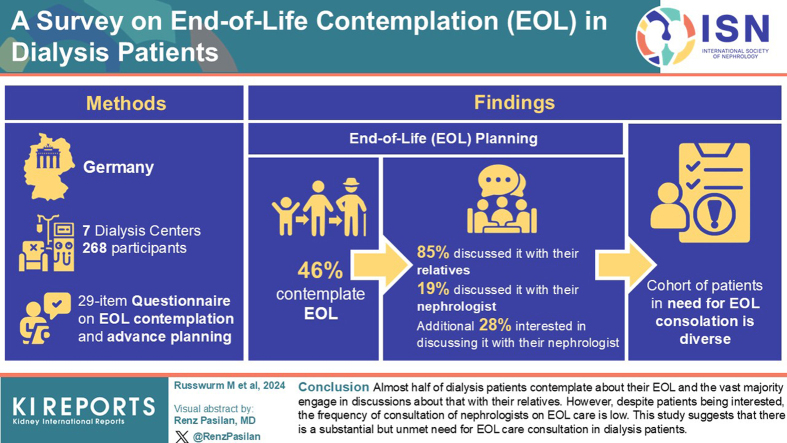

See Commentary on Page 2842

Patients on dialysis are confronted with high mortality rates and low quality of life.^1^ In a recent multinational cohort of incidental patients on dialysis in northern Europe, the first-year mortality was 19.3%,^2^ corroborating earlier data from the DOPPS registry.^3^ In elderly patients on dialysis, the outcome is even worse; 1-year mortality ranges from 46.9% among patients aged 65 to 74 years to over 70% in above-85-year-old incidental patients on dialysis in a recent study in the United States.^4^ Patients on dialysis have mortality risks that well exceed those of certain cancer entities, such as regional breast, prostate and kidney cancer, lymphoma, and chronic leukaemia.^5^ Importantly, as opposed to cancer patients of which at least a proportion can be cured, patients on dialysis will always die earlier than their matched counterparts from the general population, because end-stage kidneys never heal. However, palliative kidney care is not included in contemporary international or national guidelines in nephrology,^6^ or in board-certification requirements for nephrologists. In fact, patients on dialysis are more likely to die in the hospital and less likely to receive hospice care than cancer patients.^7^^,^^8^ During the final month in life, patients on dialysis spend more time in intensive care units and are more likely to receive invasive procedures, such as mechanical ventilation and resuscitation than patients with heart failure and cancer.^9^ Anticipatory or precautionary care planning is associated with a reduction in hospitalizations as well as in-hospital deaths.^10^ More detailed information about the individual care situation and wishes regarding EOL, including the desired place of death, could identify specific groups within the dialysis population as well as incentivize organizational changes to optimize care of people with chronic kidney disease. Therefore, we sought to investigate if patients on dialysis contemplate their EOL wishes, communicate them, and engage in advance care planning. This study also aimed to investigate whether there are specific features of patients that want to talk about their EOL with their nephrologists, in order to help identify patients with unmet consultation needs more easily.

## Methods

The study was approved by the ethics committee of the Department Of Human Medicine of the Philipps University of Marburg, Germany (reference number: 148/22). The patients were recruited from 7 German dialysis centers (all in the mid-German federal state of Hesse: cities of Giessen; Lich; Marburg: University dialysis center and local dialysis provider in Marburg-Cappel; Bad Nauheim; Schwalmstadt/Fritzlar and Wetzlar), and were eligible if capable of reading, writing, comprehend spoken word (in German and/or Russian), and giving informed consent to participate. For stringency and to avoid inter interrogator differences in communication with patients, all patients were interviewed by the same interrogator with trained skills in addressing sensitive issues. The main end points were prevalence of advance directives or care planning documents, frequency of contemplation about wishes for EOL care and previous conversations with relatives or nephrologists about those. The questionnaire (Supplementary File 1) in total contained 29 items related to demographical (age, sex, and nationality), socioeconomic (profession, highest educational degree, marital status, number of children, need for assistance, and housing) and medical factors (duration of renal replacement therapy, transplant waitlisting, concomitant diseases, knowledge about the underlying kidney disease, symptoms, symptom burden, and quality of life) potentially associated with the main end points. Quality of life was measured on a numerical scale from 0 to 10; with 0 being the lowest quality of life and 10 depicting highest quality of life. The interrogator went through the questionnaire with participants 1-by-1 while they were on dialysis.

### Statistical Methods

Most of the data are descriptive. At a 2-sided significance level of 5%, statistical analyses were carried out as specified in the respective data set. For parametrical data, a *t* test was performed. In case of violation of the homogeneity of variance prerequisite or when making comparison of medians, a Wilcoxon rank sum test was applied. Comparison of dichotomous data was performed using Fisher exact test. Correlations were calculated as Spearman’s rho. Missing data were omitted for analyses. Statistical analyses were carried out with GraphPad PRISM 8 software (GraphPad Inc).

## Results

### Principal Characteristics

Of the 349 identified patients on dialysis, 268 participated (recruitment of 77%). Reasons for nonparticipation were denial (73%), language barriers (21%), and cognitive incapacity (6%). The participating individuals (36% female) had a median age of 70 (IQR: 58–80) years. Nearly half of the patients were married (48%), 21% were widowed persons, and 18% were persons living on their own. The vast majority of patients (91%) identified as German nationals. Most patients lived outside nursing homes (94% vs. 6%; Table 1). However, half of patients required some sort of (nursing) support (22% by relatives, 22% by ambulatory nursing, and 6% in a nursing home). The participants had spent a median of 3 (IQR: 1–8) years on dialysis. Only a minority (31%) claimed to know the cause of their kidney failure.

**Table 1:** Demographic data

Category	*N* = 268	%
Sex (male/female)	171 / 97	64 / 36
Age, yr; median (IQR)	70 (58–80)	
<40	16	6
40–59	57	21
60–79	124	46
> 80	71	27
Nationality (German/other)	242 / 24	90 / 10
Duration of CKD G5 D, yr; median (IQR)	3 (1–8)	
Knowledge of cause of kidney disease (yes)	83	31
Comorbidities (multiple answers possible)		
Heart disease	133	50
Lung disease	25	9
Bowel disease	22	8
Nerve disease	42	16
Cancer	18	7
Marital status		
Married	129	48
Widowed	57	21
On their own	47	18
Divorced	28	10
Permanent relationship	7	3
Housing/nursing support		
Autonomous	239	89
With relatives	15	4
Assisted living	3	1
Nursing home	11	6
House mates (self-excluded)		
None	83	31
1	137	54
≥ 2	48	18
Nursing support		
None	135	50
By relatives	60	22
Ambulatory nursing	60	22
Nursing home	13	6

CKD, chronic kidney disease; IQR, interquartile range.

**Figure 1:**
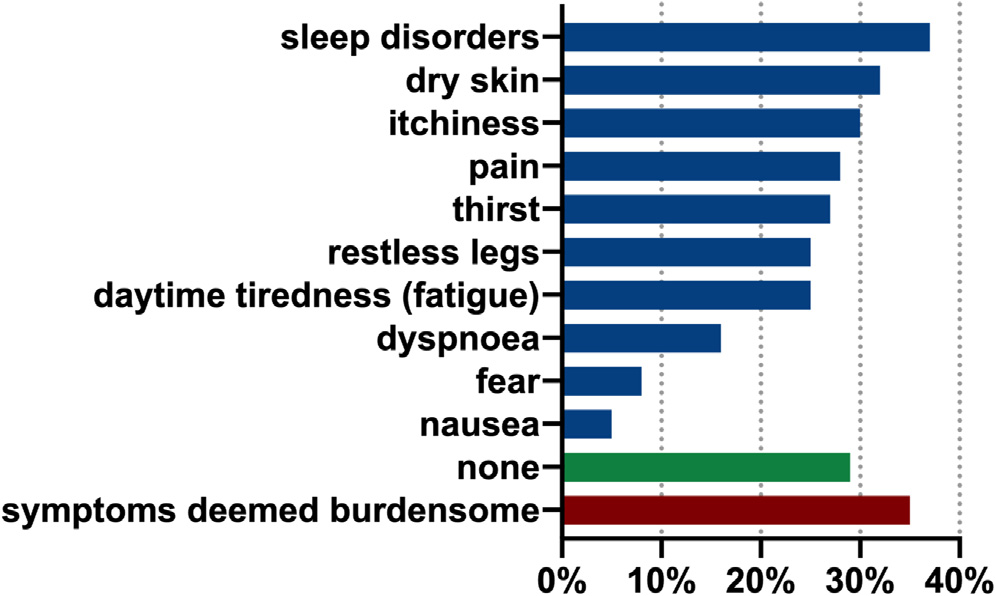
Symptoms. From top to down, symptoms according to frequency as stated by patients. Multiple answers were possible. The green bar represents percentage of patients having stated no symptoms at all. Red bar represents percentage of patients considering symptoms as being burdensome.

The median quality of life was reported to be 6 (IQR: 5–7.5). Most patients (71%) reported experiencing some kind of symptoms: sleep disorders (37%), dry skin (32%), itchiness (30%), and pain (28%) being most common; whereas dyspnea (16%), fear (8%), and nausea (5%) were the least prevalent (Figure 1). Approximately one-third of patients reported their symptoms to be relevant (35%), with daytime tiredness, dyspnea, and skin itchiness particularly being reported to be burdensome (*r*^*2*^ = 0.41, *P* < 0.001; *r*^*2*^ = 0.39; *P* < 0.001; *r*^*2*^ = 0.26, *P* = 0.013, respectively) and associated with lower quality of life (daytime tiredness: *r*^*2*^ = −0.29, *P* < 0.013; dyspnea: *r*^*2*^ = −0.25, *P* < 0.033; skin itchiness: *r*^*2*^ = −0.33, *P* = 0.005). Interestingly, the time since dialysis initiation was not associated with symptom load.

### Contemplation About EOL Wishes

**Figure 2:**
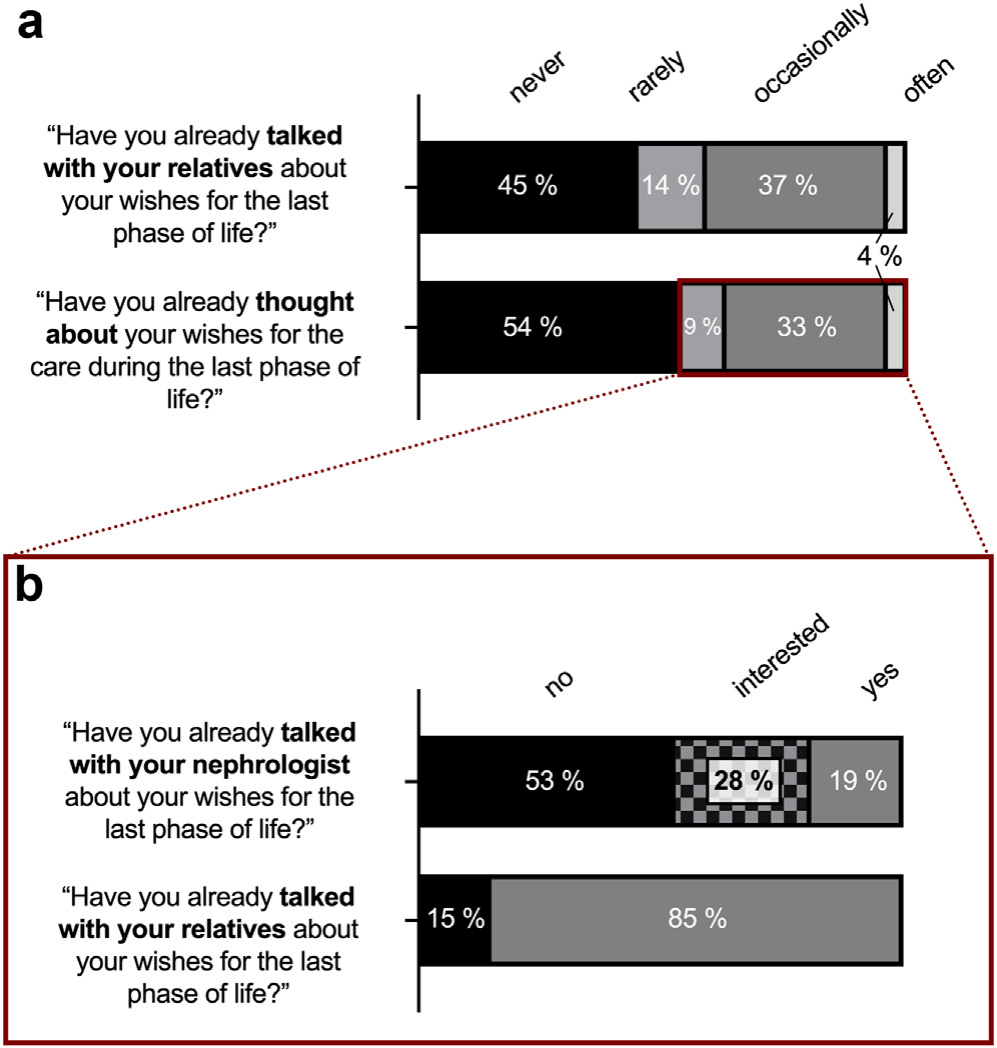
(a) Thoughts and conversations about end-of-life (EOL) wishes. Answers from patients to the respective questions in the whole (b) cohort and in those patients, who contemplate to some extent about their EOL wishes (shown in the red rectangle). Note that 81% of patients who contemplate their EOL wishes did not already talk about that with their nephrologist, but 28% of those stated to be interested in doing so.

Almost half of all patients (*n* = 123; 46%) reported to contemplate their EOL wishes to some extent (Figure 2a). Of those patients, 85% had talked about their EOL wishes with their relatives and 19% with their nephrologist (Figure 2b). The subject of EOL care has been raised with 48% of patients by their relatives, but only with 7% by the nephrologists.

An additional 28% of patients were interested in talking about EOL care opportunities with their nephrologists. In total, 46.5% of patients had or wanted to have conversations about EOL wishes with their nephrologists. Of note, there was a strong association of “being addressed by the nephrologist about EOL care” with the respective dialysis center: 58% of all patients who have been addressed by a nephrologist were from a single center. That center has had a guideline implemented demanding regular questioning of patients about their EOL wishes. With that center excluded from the analysis, 4% of patients have been addressed by the nephrologist. Interestingly, age was not associated with contemplating EOL wishes (*r*^*2*^ = 0.09, *P* = 0.14). In addition, age alone was not associated with being addressed by relatives or the nephrologist about EOL wishes (*r*^*2*^ = 0.08, *P* = 0.21). The number of hospitalizations within the past year and contemplation about EOL wishes did, however, weakly correlate with being addressed by a nephrologist about EOL wishes (*r*^*2*^ = 0.16, *P* = 0.011 and r^2^ = 0.16, *P* = 0.026, respectively).

### Advanced Directives, Care Planning, and Knowledge About Palliative Care

**Figure 3:**
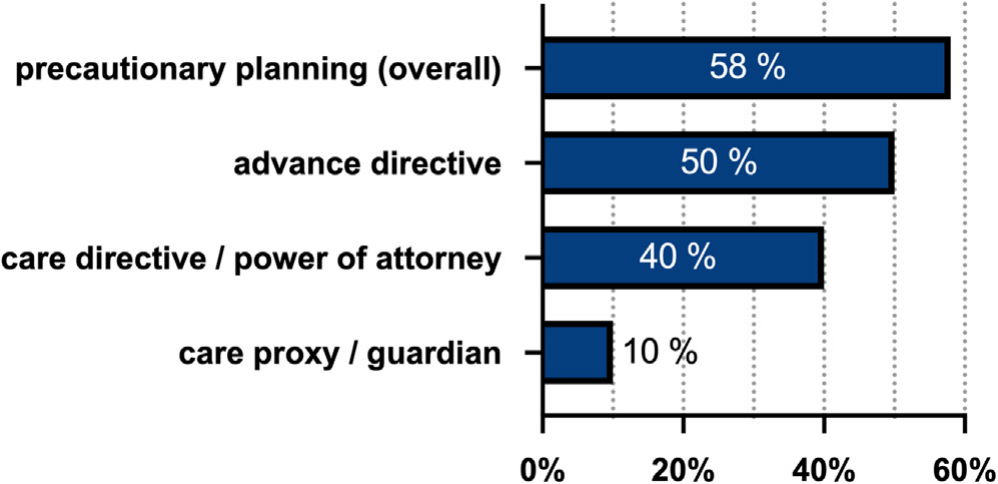
Precautionary planning. Percentage of patients having issued precautionary planning (overall and in detail) or have a care proxy.

Of all patients, 58% had some kind of precautionary planning implemented (Figure 3). Whereas 67% claimed to know what a hospice is, 52% reported to know what palliative care is. Interestingly, knowledge about palliative care and hospice was inversely correlated with age (*r*^*2*^ = −0.16, *P* = 0.008 and *r*^*2*^ = −0.30, *P* < 0.0001, respectively). However, age was associated with implementation of advance directives and care directives (*r*^*2*^ = 0.36, *P* < 0.0001 and *r*^*2*^ = 0.27, *P* < 0.0001, respectively).

### Preferred Place of Death Place of Death and Respective Notifications

Of all patients, 64% wanted to die at home. However, 25% of patients did not report a preference. The remainder of patients wanted to die in the hospital (4%), at the nursing facility (3%), at their relative’s house (2%), or at a hospice or palliative ward (2%). A minority of patients had their relatives informed about their favored place of death (38%), fewer patients reported the place of death in their advance directive (21%); basically none informed their nephrologist (1%).

### Patient Groups Possibly Discriminated Against

In our data set, between persons who identified as male and those who identified as female, we did not identify differences in EOL contemplations, precautionary planning efforts, or discussions about EOL wishes with relatives or nephrologists. However, we found that non-German self-identification (*n* = 24 persons, 9% of the cohort) was inversely correlated with issued advance directives (*r*^*2*^ = −0.210, *P* = 0.001) knowledge about hospice and palliative care (*r*^*2*^ = −0.198, *P* = 0.001; *r*^*2*^ = −0.272, *P* < 0.001; respectively) and thoughts and talks about EOL wishes (*r*^*2*^ = −0.165, *P* = 0.006; *r*^*2*^ = −0.226, *P* < 0.0001). There was no association of educational level with any items investigated.

## Discussion

This cross-sectional multicentric regional study assessed the frequency of EOL contemplation among patients on hemodialysis from mostly outpatient dialysis centers in mid-Germany. We found that approximately half of patients on dialysis dwell on their personal EOL demands for care. It was noteworthy that half of patients on dialysis were not concerned with their wishes for EOL care. Interestingly, we did not find an increased frequency of EOL contemplation in elderly patients, yet elderly patients were more likely to report to have an advance directive. However, we found that only half of patients on dialysis issued precautionary planning. These findings fit well in the body of literature,^11^ where the frequency of advance directives was demonstrated to be consistently lower among patients on dialysis than in persons with other serious illnesses.^12^ Interestingly, a recent study in the US found that a low frequency of precautionary planning was associated with overly optimistic life expectancy among patients on dialysis^13^, which might be a result of a hesitancy of nephrologists to share information on the trajectories of severe kidney disease.^14^^,^^15^

Consistently, among patients concerned about their EOL, we identified a gap between the frequency that patients engage in discussions about their EOL with relatives as opposed to their nephrologists. In fact, patients were four times more likely to have talked with their relatives about EOL care than with their nephrologist. Conceivably, not every issue concerning the EOL is in the scope of professional medical care; thus, the content of such communications might likely differ. This concept is corroborated by our finding that half of patients contemplating EOL care do not want to talk with their nephrologist. This is why we asked whether patients who did not yet talk to their nephrologist about EOL care had a wish to do so. We found that more patients wanted to talk with their nephrologist about EOL care, than already did. Taken together, almost half of patients on dialysis contemplating EOL care seek consultation from their nephrologist, yet a minority of those patients received it. The percentage of EOL care consultation among patients on dialysis improved as compared to earlier data,^16^ but remained low. This is in line with a recent German study pointing out that structured palliative care is delivered in only 20% of dialysis centers investigated.^17^ Taken into context, one has to acknowledge, that in a survey of dermatological and urological cancer patients, where the similar questionnaire was used, the unmet consultancy need between patients who already had conversations about EOL planning and those who wanted to have them was much higher.^18^^,^^19^ These findings point to a certain awareness of palliative care needs in nephrological practice. However, as of date, board certification for nephrology does not require palliative care education or rotation, neither in Germany^20^ nor in the US (American Board of Internal Medicine/ABIM)^21^ or UK (European Specialty Examination in Nephrology/ESENeph). Accordingly, in a nationwide cohort study of nephrology fellows in the US, the quality of palliative care education did not improve in a decade (2003–2013) and overall was considered low.^22^

In addition, our study finds that frequency of nephrological EOL care consultation is directly linked with institutional requirements to evaluate patients state of mind about EOL care and offering consultancy, as demonstrated in the high frequency of EOL consultation in the center with regular questioning of patients implemented. Thus, our data not only indicate that there is an unmet need for EOL care consultation among patients on hemodialysis, but that regular questioning of patients about EOL care might effectively identify those in need. This is especially important, as we could not identify distinctive characteristics of patients contemplating EOL or demanding consultation. These findings corroborate earlier data, that patients expect nephrologists to initiate and guide precautionary planning.^23^ Our data on symptoms, symptom burden, and quality of life of patients on dialysis are in line with previous reports and match the characteristics found in cancer patients.^24^ This might serve as another argument in favor of acknowledging palliative care needs for patients on dialysis and might by implication reason reflections on shortcomings of “contemporary” palliative care for patients on dialysis. These shortcomings, arguably manyfold, ranging from legal uncertainty, understaffing, and lack of financial resource to lack of expertise, were recently highlighted by articles interrogating nephrologists and dialysis nursing staff on their views on barriers to integrated palliative care for their patients.^17^^,^^25^ Largely missing in the literature is the important active part that dialysis nursing staff might play in the palliative care network. Here again, education and training emerge as the key factors in a recent qualitative study.^26^

One main rational to engage in advance care planning in palliative care patients is the avoidance of “overtreatment,” unwanted treatment at the EOL, and to maintain autonomy in medical emergencies. In addition, written advance directives may substantially ease the burden of “proxy-responsibility” for relatives^27^^,^^28^ and for other care givers, including nurses and physicians. Naturally, it is conceivable that fear of death and dying can be relieved if certain scenarios are readily talked through.^29^

Given the beforementioned advantages of precautionary planning and conversations, it is well-recognized that there are multiple barriers to frank communication about death and dying between physicians, patients and patients’ relatives, and care givers, which might be identified as being of emotional, cognitive, and cultural texture.^30^ In addition, the impact of religious beliefs on demands for EOL care by patients on dialysis should not be neglected.^31^ In that latter study, for example, participants who identified as having strong religious beliefs were more likely to prefer cardiopulmonary resuscitation. In our study, we found that patients who did not identify as German had lesser knowledge about palliative and hospice care and less frequently talked about EOL wishes, both with relatives and the treating physicians. These data highlight the importance of recognizing vulnerable patient subgroups with special demands within the dialysis collective. Most interestingly, given the possible value of early palliative care consultation demonstrated,^32^ among patients on dialysis, there seems to be a disconnect between expressed values that were mostly comfort-centered, and patients’ engagement in precautionary planning and EOL care, which reflected a focus on longevity.^33^ Accordingly, patients’ values and certain aspects of EOL care might not align with “Do not resuscitate” - code status: a recent study found that a minority (17%) of patients on dialysis would refrain from cardiopulmonary resuscitation; however, a minority of those patients issued advance directives. As in most circumstances a “no-code-status” is processed as maximum therapy, those patients are specifically vulnerable to therapies and procedures not matching their predefined values.^34^ In the same study, 1 out of 4 patients who favored cardiopulmonary resuscitation, did not want mechanical ventilation, which according to the authors may point to a lack of patients’ education on implications of cardiopulmonary resuscitation.

Taken together, patient education and subsequent informed precautionary planning is key in aligning therapeutic and care attempts to patient wishes, demands, and values. One important step in the process is initiating (and frequently repeating) conversations on EOL care with patients with serious diseases. Our study elaborates on the implementation of these conversations in regular nephrology care in 7 dialysis centers and finds that the majority of patients engaged in discussions about EOL care with relatives, but not with nephrologists. Importantly, our data show that there is a substantial proportion of patients who want to discuss questions of EOL care with their nephrologist. We could not find a set of characteristics that reliably identifies patients with an unmet consultation need. Thus, regularly questioning of patients on hemodialysis about EOL wishes and, consequently, implementing EOL care skills in the nephrology training catalogue seems advisable.

### Limitations

This study was conducted in Germany; thus, results might not reflect the specific situation in other national contexts, for example, with different cultural and religious backgrounds of patients and care takers. This is especially important because we did not investigate religious beliefs, which were shown to significantly influence medical decision making strategies.^31^ Naturally, we lack information on the 23% of patients who were not eligible or denied participation in the study. Furthermore, Likert-like scales pose an inherent risk of reporting bias. We intended to control that risk with 1-on-1 interview; however, by design, interrogation surveys on their own right might be subject to substantial social desirability bias. This is especially true for studies in which patients are confronted with interrogators and not filling out a form anonymously. We thoughtfully chose this approach, to ensure comprehension of questions and completeness of data. Lastly, in this study we cannot confirm accuracy of patients’ responses because there was no questioning of nephrologists or review of medical records undertaken.

## Conclusion

This study examined the patient-reported *status quo* of EOL thoughts and planning among patients on hemodialysis. Our data show, that half of patients on dialysis dwell on the EOL and the vast majority consult with relatives about wishes concerning the EOL, but only a minority does consult with the nephrologist. There is a substantial proportion of patients who want to talk with their nephrologist about EOL wishes but did not do so. Those patients might be accessible to initiating conversations by the nephrologist about EOL care, thus empowering patients and care givers to align EOL care to individual patients’ wishes and values.

## Disclosure

All authors declared no competing interests.

## Supplementary Material

Supplementary File (PDF) Questionnaire.

## Footnotes

Supplementary File (PDF)
Questionnaire
