# Corrigendum to “A Survey on End-of-Life Contemplation Among Patients on Dialysis” [*Kidney International Reports* Volume 9, Issue 10, October 2024, Pages 2981-2987]

**DOI:** 10.1016/j.ekir.2026.107047

**Published:** 2026-08-20

**Authors:** Martin Russwurm, Anetta Rabaev, Joachim D. Hoyer, Christian S. Haas, Christian Volberg, Philipp Russ

**Affiliations:** 1 Division of Nephrology, Centre for Internal Medicine, University Hospital Marburg, Philipps University Marburg, Marburg, Germany; 2 Institute of Pharmacology, Philipps University Marburg, Marburg, Germany; 3 Department of Anesthesiology and Intensive Care Medicine, University Hospital Marburg, Philipps University Marburg, Marburg, Germany; 4 Research Group Medical Ethics, Faculty of Medicine, Philipps University Marburg, Marburg, Germany

The authors regret that the original publication in which the questionnaire underlying the interviews in their study was inadvertently omitted from the references. Two publications were cited in which the questionnaire had subsequently been applied; however, the primary validation study itself was not referenced. The complete reference is now shown below:

Pedrosa AJ, Feldmann S, Klippel J, et al. Factors associated with preferred place of care and death in patients with Parkinson’s disease: a cross-sectional study. *Journal of Parkinson’s Disease*. 2024;14(3):589-599.

DOI of original article: https://doi.org/10.1016/j.ekir.2024.07.035

