# Supplementary material for: A Survey on End-of-Life Contemplation Among Patients on Dialysis
Source: Kidney Int Rep. 2024 Aug 3;9(10):2981–7. doi: 10.1016/j.ekir.2024.07.035 (PMC11489826; doi:10.1016/j.ekir.2024.07.035)
Supplement: Supplementary File (PDF) — Questionnaire. [file mmc1.pdf]

Patient ID:

Date:

Questionnaire

Please answer the following questions.

1. **What is your sex?**
   - o female
   - o male
   - o other

2. **How old are you?**

3. **How long do you suffer from kidney disease?**

4. **How old have you been, when you started dialysis?**

5. **Are you aware of your specific kidney disease?**
   - o Yes, my kidney disease is:__________________
   - o no

6. **Do you suffer from one or more of the following serious conditions?**
   - o Heart disease *(e.g. heart failure, previous heart attack / infarction)*
   - o Lung disease *(e.g. Chronic obstructive pulmonary disease, asthma)*
   - o Intestinal disease *(e.g. chronic inflammatory bowel disease)*
   - o Nerve diseases *(e.g. stroke, Parkinsons disease)*
   - o Cancer

7. **Your nationality is**
   - o german
   - o other:_______________________

8. **What is your marital status?**
   - o single / unmarried
   - o steady relationship
   - o married
   - o widowed
   - o divorced

Patient ID:

9. Do you have children?

o yes, I have ________ children
o no

10. Where do you live?

- o Own household
- o With my children / relatives
- o Assisted living
- o Nursing home (proceed with question 15)
- o other: _______________________

11. How many persons are permanently living in your household (yourself included)?

12. Do you have a hobby?

- o sports
- o meditation
- o other:__________________________
- o nein

13. Do you receive/require nursing support?

- o no
- o yes by relatives
- o yes by a professional nursing service at home
- o yes, in a nursing home
- o yes, by:__________________________

14. What is your highest degree of education?

- o _________________________

15. Have you issued the following documents (multiple answers possible)?

- o Living will
- o Power of attorney
- o Other documents to state my wishes for end-of-life care: ______________________________
- o I do not know

15b. Do you have a proxy / guardian?

- o yes
- o no

16. Do you know what palliative care is?

- ○ yes
- ○ no
- ○ I do not know

17. Do you know what a hospice is?

- ○ yes
- ○ no
- ○ I do not know

18. Did you ever contemplate about your wishes for the end of life?

- ○ often
- ○ occasionally
- ○ rarely
- ○ never

19. Did you have conversations with your relatives / loved ones about your wishes for the end of life?

- ○ often
- ○ occasionally
- ○ rarely
- ○ never

20. Have you already been addressed by anybody about your wishes for the end of life?

- ○ often
- ○ occasionally
- ○ rarely
- ○ not yet

21. If you had been addressed about your wishes for the end of life, by whom? (multiple answers possible)

- ○ Significant other
- ○ My child / children
- ○ friends
- ○ general practitioner
- ○ nephrologist
- ○ other: ______________________________
- ○ I was never adressed

22. Did you discuss a plan with your nephrologist, if your medical situation deteriorates?

- ○ yes
- ○ no
- ○ I do not know

23. If you did not discuss a plan with your nephrologist, would you be interested to have such a discussion?

- yes
- no
- I do not know

24. Do you suffer from one or more of the following symptoms?

o no
o yes (multiple answers possible):

o pain
o shortness of breath
o nausea
o diarrhea
o sleeping disorder
o daytime tiredness (fatigue)
o itchiness
o dry skin
o thirst
o restless legs
o fear
o other: ________________

25. Do you consider those symptoms burdensome?

o yes
o no
o I do not know

26. Where would you most "like" to die (what is your preferred point of death)?

- At home
- Nursing home
- hospital
- palliative care ward
- hospice
- with my relatives
- other: _________________________
- I do not care

27. Did you tell that to anybody?

- yes:
  - relatives / friends
  - general practitioner
  - nephrologist
- no
- I do not know

28. If you have a living will or power of attorney, did you state your preferred point of death in that document?

- yes
- no
- I do not know

29. How often have you been in the hospital for emergencies in the last 12 months?

- ___ times
- never
- I do not know

We thank you very much for your time and wish you all the best for the future!
